# Epidemiology of *Clostridium difficile* in infants in Oxfordshire, UK: Risk factors for colonization and carriage, and genetic overlap with regional *C*. *difficile* infection strains

**DOI:** 10.1371/journal.pone.0182307

**Published:** 2017-08-16

**Authors:** Nicole Stoesser, David W. Eyre, T. Phuong Quan, Heather Godwin, Gemma Pill, Emily Mbuvi, Alison Vaughan, David Griffiths, Jessica Martin, Warren Fawley, Kate E. Dingle, Sarah Oakley, Kazimierz Wanelik, John M. Finney, Melina Kachrimanidou, Catrin E. Moore, Sherwood Gorbach, Thomas V. Riley, Derrick W. Crook, Tim E. A. Peto, Mark H. Wilcox, A. Sarah Walker, the Modernising Medical Microbiology Informatics Group (MMMIG)

**Affiliations:** 1 Nuffield Department of Clinical Medicine, Oxford University, John Radcliffe Hospital, Headington, United Kingdom; 2 National Institute for Health Research (NIHR) Oxford Biomedical Research Centre, John Radcliffe Hospital, Headington, United Kingdom; 3 Leeds Teaching Hospitals and University of Leeds, Department of Microbiology, Old Medical School, Leeds General Infirmary, Leeds, United Kingdom; 4 Public Health England (Leeds laboratory), Old Medical School, Leeds General Infirmary, Leeds, United Kingdom; 5 Microbiology Laboratory, John Radcliffe Hospital, Headington, United Kingdom; 6 Department of Microbiology, Medical School, Aristotle University of Thessaloniki, University Campus, Thessaloniki, Greece; 7 Tufts University School of Medicine, Boston, Massachusetts, United States of America; 8 Microbiology and Immunology, School of Pathology and Laboratory Medicine, The University of Western Australia, Crawley, Western Australia, Australia; Cleveland Clinic, UNITED STATES

## Abstract

**Background:**

Approximately 30–40% of children <1 year of age are *Clostridium difficile* colonized, and may represent a reservoir for adult *C*. *difficile* infections (CDI). Risk factors for colonization with toxigenic versus non-toxigenic *C*. *difficile* strains and longitudinal acquisition dynamics in infants remain incompletely characterized.

**Methods:**

Predominantly healthy infants (≤2 years) were recruited in Oxfordshire, UK, and provided ≥1 fecal samples. Independent risk factors for toxigenic/non-toxigenic *C*. *difficile* colonization and acquisition were identified using multivariable regression. Infant *C*. *difficile* isolates were whole-genome sequenced to assay genetic diversity and prevalence of toxin-associated genes, and compared with sequenced strains from Oxfordshire CDI cases.

**Results:**

338/365 enrolled infants provided 1332 fecal samples, representing 158 *C*. *difficile* colonization or carriage episodes (107[68%] toxigenic). Initial colonization was associated with age, and reduced with breastfeeding but increased with pet dogs. Acquisition was associated with older age, Caesarean delivery, and diarrhea. Breastfeeding and pre-existing *C*. *difficile* colonization reduced acquisition risk. Overall 13% of CDI *C*. *difficile* strains were genetically related to infant strains. 29(18%) infant *C*. *difficile* sequences were consistent with recent direct/indirect transmission to/from Oxfordshire CDI cases (≤2 single nucleotide variants [SNVs]); 79(50%) shared a common origin with an Oxfordshire CDI case within the last ~5 years (0–10 SNVs). The hypervirulent, epidemic ST1/ribotype 027 remained notably absent in infants in this large study, as did other lineages such as STs 10/44 (ribotype 015); the most common strain in infants was ST2 (ribotype 020/014)(22%).

**Conclusions:**

In predominantly healthy infants without significant healthcare exposure *C*. *difficile* colonization and acquisition reflect environmental exposures, with pet dogs identified as a novel risk factor. Genetic overlap between some infant strains and those isolated from CDI cases suggest common community reservoirs of these *C*. *difficile* lineages, contrasting with those lineages found only in CDI cases, and therefore more consistent with healthcare-associated spread.

## Introduction

*Clostridium difficile* remains one of the commonest causes of nosocomial infective diarrhea in high-income countries[1], increasing costs and morbidity[2]. Community-associated cases increasingly contribute to disease burden[3]. Children <1 year of age are commonly colonized with toxigenic and non-toxigenic *C*. *difficile* (cross-sectional prevalence 35–40%), decreasing to adult rates of ~3% by ~8 years[4], with generally similar multi-locus sequence types (STs)/ribotypes as in adult *Clostridium difficile* infections (CDI)[5]. A notable exception is the lack of any observed colonization by epidemic ST1(027) in healthy asymptomatic children[5, 6], although it is found in hospitalized children[7]. Previously identified risk factors for pediatric *C*. *difficile* colonization or carriage include mode of delivery, age, breastfeeding/nutrition, previous antibiotic use, and the environment in which the child is residing (e.g. hospitalized, out-patient)[8–11]; these have, however, been relatively small studies.

Given the high prevalence of *C*. *difficile* colonization and carriage in children, we investigated predominantly healthy children ≤2 years old in Oxfordshire, UK, with limited healthcare contact, to determine risk factors for colonization with and acquisition of toxigenic and non-toxigenic *C*. *difficile*. We used WGS, as the most discriminatory typing tool available[12], to characterize *C*. *difficile* strains isolated, their population structure, and their molecular epidemiology within individual hosts over time, and compared these with strains isolated from Oxfordshire CDI cases.

## Materials and methods

### Study design

All children ≤2 years of age (denoted infants) living in Oxfordshire, UK (population ~653800 people, ~16700 infants[13]), were eligible, provided signed, informed consent was obtained from their parent/legal guardian. Between March 2010-November 2012 infants were recruited from local breastfeeding advice clinics/cafés, well-baby clinics (offering routine postnatal care), nurseries (childcare centers taking children from 4–6 weeks of age) and local vaccine studies. A minority of infants (16%) was recruited from the John Radcliffe Hospital, Oxford (emergency department [<48 hours of admission, 14% of participants]; hospital wards [≥48 hours of admission, 2%]). We chose not to sample newborn nurseries as these were likely to reflect reservoirs of healthcare-associated strains and our study was focused on predominantly healthy, community-dwelling infants.

Caregivers provided an initial infant fecal sample and completed a questionnaire covering putative risk factors for colonization, including those identified in previous infant *C*. *difficile* colonization and carriage studies (Supporting information [a]). Caregivers of infants ≤6 months of age were asked if they would additionally enroll their infant in a longitudinal study, involving monthly collection of fecal samples and questionnaire data for 9 months (last follow-up July 2013). Data collected longitudinally included factors recorded at enrolment that could vary over time (Supporting information [a]).

Given the variability in “normal” infant stooling patterns, we adopted a pragmatic approach to this definition: Infants were pre-defined as “symptomatic” if they had more frequent/looser stools according to their caregiver, and “asymptomatic” if their stool pattern was considered normal by the caregiver. They were considered colonized (or carriers) if one (or more consecutive) isolate(s) cultured from their fecal sample was confirmed as *C*. *difficile* by WGS. For the longitudinal cohort, “acquisition” included a change from either uncolonized to colonized or a change of *C*. *difficile* strain, as defined by WGS. The study was designed to enroll 450 infants (≥150 in “symptomatic”,”asymptomatic” groups) to provide ≥80% power to detect differences of 15% in colonization prevalence between the groups (two-sided α = 0.05). The study was approved by the Oxford NHS Research Ethics Committee (OxREC C 09/H0606/80).

### Genetic comparisons with sequenced Oxfordshire *C*. *difficile* infection-associated isolates

Whole genome sequences from infant *C*. *difficile* colonization and carriage were compared with each other, and with consecutive strains isolated from symptomatic Oxfordshire CDI cases and potential excretors (denoted Oxfordshire symptomatic patients) identified as part of routine clinical testing (September 2006-September 2013)[14, 15]. Samples from patients with diarrhea were submitted from hospital inpatients and primary care for *C*. *difficile* testing. To March 2012, only enzyme-immunoassay (EIA) positive samples were identified; from April 2012 glutamate dehydrogenase (GDH) testing was used as part of a two-step testing process, and GDH-positive samples were processed for sequencing regardless of whether they were EIA-negative (presumed *C*. *difficile* colonized, with diarrhea of another cause) or EIA-positive (CDI cases). As part of a *C*. *difficile* diagnostic study[16], strains not expressing toxin were also identified by culture between December 2010 and September 2011 inclusive. 21/2259 [0.9%] cases where age was known were ≤2 years of age.

### Laboratory methods and WGS

DNA was extracted from single colony sub-cultures incubated for 48 hours on Columbia blood agar[17]. Extracts were sequenced using Illumina HiSeq 2000 technology (Illumina Inc, San Diego, CA), generating 100 base-pair paired-end reads. Reads were mapped to the *C*. *difficile* reference genome 630 (Genbank: AM180355.1) and sequences compared using single nucleotide variants (SNVs). SNV differences between samples were obtained from maximum likelihood phylogenetic trees, initially constructed with PhyML[18], (generalized time reversible substitution model, “BEST” tree topology search operation option), and adjusted to remove recombining regions using ClonalFrameML[19] (default parameters)(Supporting information[b]). Reads were also *de novo* assembled using Velvet/VelvetOptimiser[20], and the presence/absence of toxin genes *tcdB* and/or *tcdA* (toxigenic *C*. *difficile*) and multi-locus sequence type determined using BLASTn.

### Rates of evolution during infant *C*. *difficile* carriage

Sequences from the first and last samples for infants carrying the same *C*. *difficile* strain were compared (n = 70), together with sequences from adults with on-going or recurrent CDI (n = 145)[14], to determine if rates of *C*. *difficile* evolution in infants differed from adults. To estimate evolutionary rates, SNVs were assumed to arise as the combination of a time-dependent Poisson process, representing evolution, and a time-independent Poisson process, representing within-host diversity/assay variation[21]. This model does not account for time spent in spore form; however, given that this likely reflects a state with slowed evolutionary rates, our estimates of genetic similarity were anticipated to be conservative.

#### Metrics of genetic relatedness

Pairs of sequences varying by ≤2 SNVs were considered sufficiently closely related to be compatible with recent direct transmission/acquisition from a common source, given rates of *C*. *difficile* evolution and within-host diversity[14]. Pairs of sequences varying by 0–10 SNVs were considered related through a shared common ancestor sometime during or shortly before the study (~5 years evolution). Pairs of sequences varying by >10 SNVs were considered genetically distinct.

### Statistical methods

Data collected at baseline (for the colonization analysis) included factors whose effects were considered important to investigate in multivariable models together with potential confounders: date of birth, gender, birth history (mode of delivery, location of delivery, gestational age, birth weight), exposure to hospital/long-term healthcare facilities (length of stay in hospital after birth, date and details of other hospital admissions since birth), relevant medical history (current diagnoses, past episodes of illness), regular medications including antibiotics, nutritional factors (current breastfeeding, formula milk or solid food intake), presence of pets/animals at home, whether anyone in the family was working in healthcare, siblings and age of siblings in the household, non-parental carers, and travel abroad in the last year.

Data collected longitudinally were all those factors collected at enrolment that could vary over time, namely relevant medical history (new medical problems, visits to GP or hospital), regular medications including antibiotics, nutritional factors (current breastfeeding, typical daily meals), presence of pets/animals at home, whether anyone in the family was working in healthcare, non-parental carers, and travel abroad, all since the last sample was taken.

When named, administered antibiotics were categorized as high risk for *C*. *difficile* (co-amoxiclav, cephalosporins, ciprofloxacin) or not[22, 23]; when not named, they were categorized as unknown risk. All unnamed antibiotics were assumed to be systemic (as 93% of named antibiotics were systemic). Topical antibiotics were not considered in analysis.

Univariable logistic regression was used to pre-select variables for multivariable analyses using a significance threshold of p≤0.2, allowing for non-linearity in continuous variables (i.e. age, gestation, weight at birth, length of hospital stay after birth, number of medical problems, number of times admitted to hospital, length of stay for hospital admissions) using fractional polynomials (Supporting material[c]). Multivariable logistic and Cox regression were used to identify independent risk factors for initial colonization with *C*. *difficile* and acquisition of distinct strains using backwards elimination (exit p = 0.05) on factors pre-selected above, and accounting for non-linearity in continuous variables using multivariable fractional polynomials. The final models were refitted in “complete cases” for the selected factors or interactions if p<0.05 (Supporting material[c]). Heterogeneity between risk factors for toxigenic and non-toxigenic strains was assessed using multivariable multinomial regression and stacked regression for colonization and acquisition respectively. Statistical analyses were performed in Stata, version 13.1(StataCorp LP, Texas, USA).

## Results

**Fig 1:**
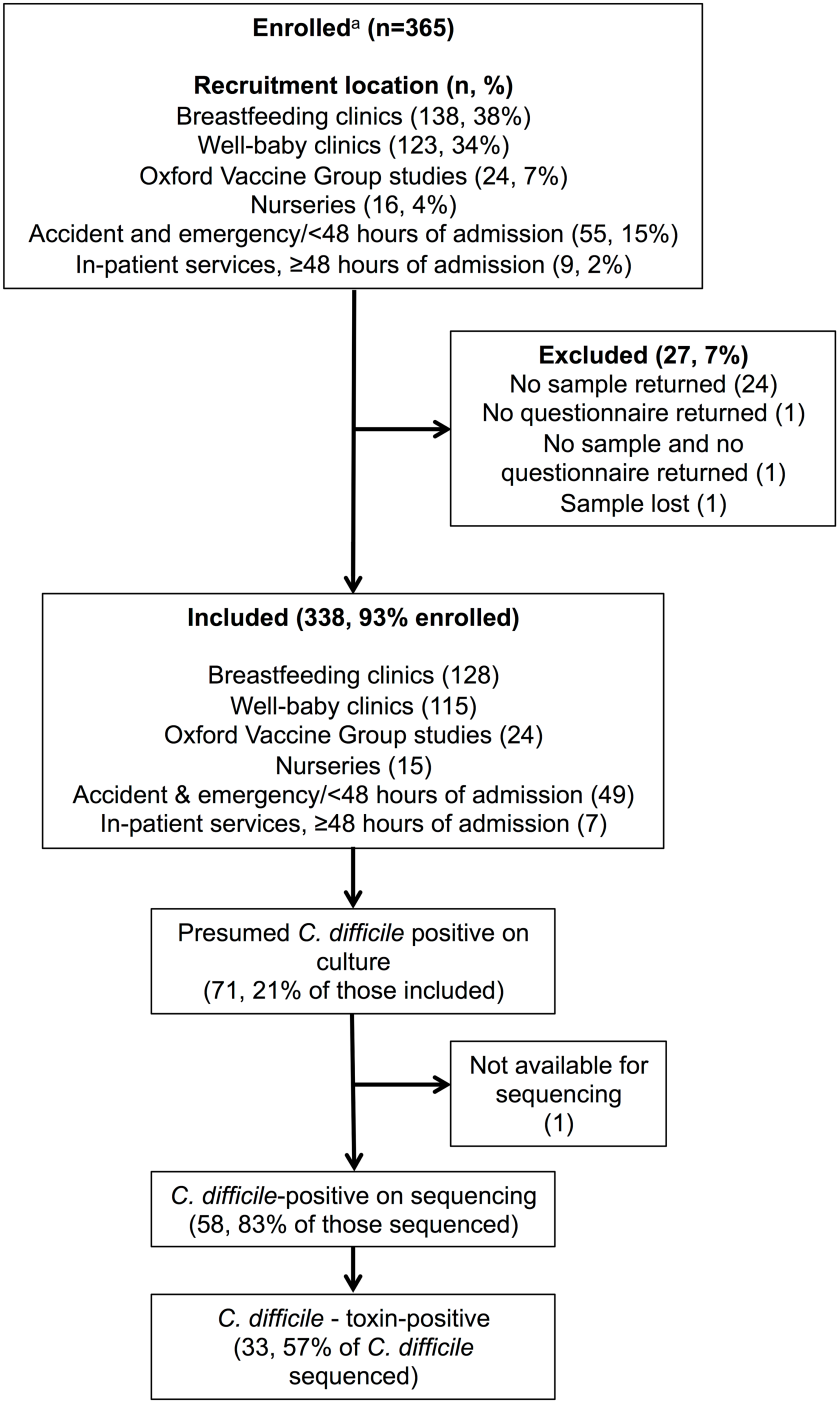
Participant disposition. ^a^ The total numbers of individuals screened for recruitment was not recorded.

365 infants were enrolled; 26 failed to return either a sample/questionnaire, and one sample was lost, leaving 338 infants, aged median (IQR) 4.4(1.6–8.7) months, for analysis(Fig 1). Only seven (2%) had hospital-onset diarrhea (≥48h after admission); all other participants (98%) were from the community.

### Risk factors for *C*. *difficile* colonization at enrolment

Overall 58(17%) infants were colonized with *C*. *difficile* at enrolment. 18 factors were associated with *C*. *difficile* colonization with p<0.05 in the univariable analyses(S1 Table); age, breastfeeding and pet dogs were independently associated(Table 1). Colonization prevalence increased to ~11 months of age and then decreased(S1 Fig), and was independently lower in mixed formula/breastfed and breastfed versus non-breastfed infants. Infants in households with dogs had significantly higher colonization rates; the protective effect of breastfeeding was attenuated in these households(interaction p = 0.02, S2 Table). Adjusting for these factors (and the interaction), there was no additional effect on colonization status of an infant having more frequent/looser versus normal stools (odds ratio [OR] = 1.19, 95%CI [0.56–2.55], p = 0.65), although power to detect an independent effect was reduced (fewer infants with more frequent/looser stools were recruited than planned, n = 61).

**Table 1:** Independent risk factors for *Clostridium difficile* colonization at enrolment.

	Overall n (col %) or median (IQR)	*C*. *difficile* positive (all strains), n = 58			Hetero-geneity of effect in non-toxigenic versus toxigenic	Presence of non-toxigenic strains, n = 25			Presence of toxigenic strains, n = 33		
Risk factor		n (row %) or median (IQR)	Odds ratio (95% CI)	p	p	n (row %) or median (IQR)	Odds ratio (95% CI)	p	n (row %) or median (IQR)	Odds ratio (95% CI)	p
**Age (months)**	4.4 (1.6, 8.7)	8.0 (5.2, 11.8)	Non-linear*	<0.001	0.48	8.7 (6.1, 10.7)	Non-linear*	0.001	7.6 (5.2, 12.6)	Non-linear*	0.03
2	122 (36%)**	8 (7%)	0.36 (0.21, 0.61)			3 (2%)	0.27 (0.12, 0.60)		5 (4%)	0.49 (0.25, 0.97)	
4	53 (16%)	3 (6%)	0.59 (0.45, 0.78)			1 (2%)	0.52 (0.34, 0.78)		2 (4%)	0.70 (0.50, 0.99)	
6	57 (17%)	6 (11%)	1.00 (ref)			2 (4%)	1.00 (ref)		4 (7%)	1.00 (ref)	
9	40 (12%)	16 (40%)	1.77 (1.28, 2.46)			7 (18%)	2.01 (1.23, 3.27)		9 (23%)	1.43 (0.94, 2.19)	
12	31 (9%)	12 (39%)	1.98 (1.18, 3.32)			8 (26%)	2.17 (0.97, 4.84)		4 (13%)	1.44 (0.74, 2.81)	
15	18 (5%)	8 (44%)	1.17 (0.58, 2.37)			3 (17%)	1.00 (0.28, 3.52)		5 (28%)	0.89 (0.37, 2.13)	
18	17 (5%)	5 (29%)	0.32 (0.09, 1.13)			1 (6%)	0.17 (0.02, 1.74)		4 (24%)	0.31 (0.07, 1.35)	
**Nutrition**											
No breastfeeding	124 (37%)	41 (33%)	1.00 (ref)			15 (12%)	1.00 (ref)		26 (21%)	1.00 (ref)	
Mixed feeding	103 (30%)	14 (14%)	0.40 (0.19, 0.83)	0.01	0.22	8 (8%)	0.70 (0.25, 1.94)	0.49	6 (6%)	0.30 (0.11, 0.82)	0.02
Breastfeeding only	111 (33%)	3 (3%)	0.13 (0.03, 0.47)	0.002	0.23	2 (2%)	0.31 (0.05, 1.73)	0.18	1 (1%)	0.06 (0.01, 0.50)	0.01
Pet dog***	58 (17%)	21 (36)%	3.06 (1.47, 6.38)	0.003	0.80	9 (16%)	2.77 (1.02, 7.56)	0.05	12 (21%)	3.23 (1.30, 8.04)	0.01
**Ever taken systemic antibiotics**	83 (25%)	23 (28%)	Not selected in model for colonization with any *C*. *difficile* strain		0.02	5 (6%)	0.58 (0.19, 1.82)	0.35	18 (22%)	2.76 (1.15, 6.60)	0.02
**Child-minder**	14 (4%)	5 (36%)	Not selected in model for colonization with any *C*. *difficile* strain		-	5 (36%)	6.35 (1.59, 25.39)	0.01	0 (0%)	No cases	-

* see S1 Fig.

** n (%) are the numbers of infants aged zero-two months, two-four months etc. Odds ratios for age effect are calculated from the best-fitting fractional polynomial function using 6 months as the reference category (and are not based on categorized age-groups which are merely shown for reference).

*** Although pet dogs were identified using backwards elimination, similar model fit and effect was found when substituting dogs with cats (main model: OR = 1.48 p = 0.26 for cats, difference in Akaike Information Criteria: 259(cats)-252(dogs) = 7). See S1 Table for univariable results.

Approximately half the colonized infants (33, 10% overall) had toxigenic *C*. *difficile*. There was no evidence that the effect of age, breastfeeding or pet dogs differed for toxigenic versus non-toxigenic *C*. *difficile* (heterogeneity p>0.2). However, previous use of systemic antibiotics independently increased the colonization risk by a toxigenic (OR = 2.76, [1.15–6.60]) but not a non-toxigenic strain (heterogeneity p = 0.02), and having a child-minder (providing non-institutional childcare, typically with several other children) increased the colonization risk by a non-toxigenic strain (OR = 6.35, [1.59–25.39])(Table 1).

### Risk factors for acquiring a new *C*. *difficile* strain

127(38%) infants contributed longitudinal sample(s) with questionnaire data (median [IQR] follow-up 8 [7–8] months, 1112 samples), and were a median (IQR) 2.5 (1.0–4.1) months old at enrolment, with lower baseline colonization (11/127, 9%). Enrolment strains were carried for median (IQR) 4 (2–9) months. 77(61%) infants acquired a new strain post-enrolment, and 25(32%) acquired ≥2 new strains. Older age at enrolment (consistent with S1 Fig), Caesarean delivery, and diarrhea since the last sample were independently associated with increased risk of acquiring a new strain (S3 and S4 Tables). Current mixed formula/breastfeeding or exclusive breastfeeding and pre-existing *C*. *difficile* colonization independently reduced acquisition risk.

62(49%) and 28(22%) infants acquired a toxigenic *C*. *difficile* and non-toxigenic *C*. *difficile* strain, respectively. There was no evidence that the effect of age, delivery mode, breastfeeding or previous colonization differed for acquisition of toxigenic versus non-toxigenic *C*. *difficile* (heterogeneity p>0.2; S4 Table); however, there was a weak trend towards diarrhea since the last sample having a greater effect on acquiring a new toxigenic *C*. *difficile* (heterogeneity p = 0.06). Other effects on acquisition of either toxigenic (cat ownership [p = 0.03, heterogeneity p = 0.08], childcare nursery attendance [p = 0.01, heterogeneity p = 0.23]) or non-toxigenic *C*. *difficile* (having a child-minder [p = 0.004, heterogeneity p = 0.02]) reflected additional environmental exposures (S4 Table).

### Genetic analyses and comparisons

The estimated rate of evolution was 0.88 SNVs/year (95% CI 0.35–1.40), with 0.28 SNVs (95% CI 0.15–0.41) arising from within-host diversity, with no evidence for different evolutionary rates between *C*. *difficile* from adult CDI cases and infants (heterogeneity p = 0.86)(S2 Fig, a single pair of infant sequences separated by 11 SNVs and 200 days was determined to be more likely to represent re-colonization with a new strain and excluded, as the second sequence had been previously found in other participants).

**Fig 2:**
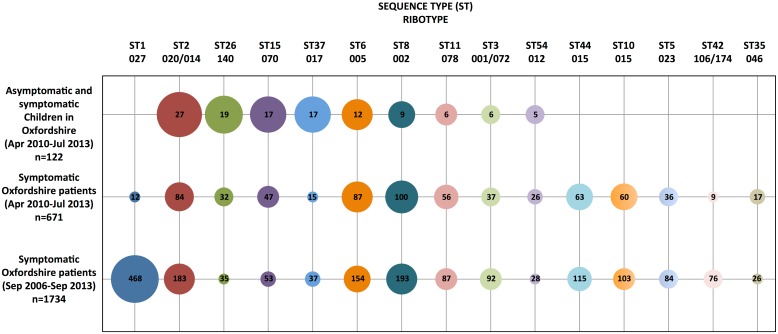
*Clostridium difficile* ST prevalence in Oxfordshire infants and symptomatic patients. ST15, ST26 and a subset of ST3 are non-toxigenic and hence were only identified in symptomatic Oxfordshire patients between December 2010 to September 2011, and from April 2012 to September 2013. Only three infant samples were obtained prior to January 2011, therefore STs from symptomatic patients are shown separately for January 2011 to September 2013 to provide a comparison of the strains circulating during the study. Circle size represents the proportion of strains per ST within an isolate collection.

Including enrolment and longitudinal infant samples, 364/1332(27%) contained *C*. *difficile*. Of 355(98%) isolates successfully sequenced, 257(72%) were toxigenic, representing 158 distinct colonization or carriage episodes in 130 infants. The most common toxigenic STs (equivalent ribotypes) were ST2(020/014/076/220), ST37(017), ST6(005), ST8(002) and ST11(078)(Fig 2). 3245 *C*. *difficile* sequences from 2286 symptomatic Oxfordshire patients were available for comparison, representing 2425 genetically distinct CDI/colonization episodes (September 2006-September 2013; 21/2259[0.9%] cases with age known were ≤2 years of age). There was close overlap between STs in infants and local symptomatic patients(Fig 2), with the exception of ST1(027) and ST10/44(015), both frequent in patients but absent in infants.

### Genetic relatedness between infant samples

**Fig 3:**
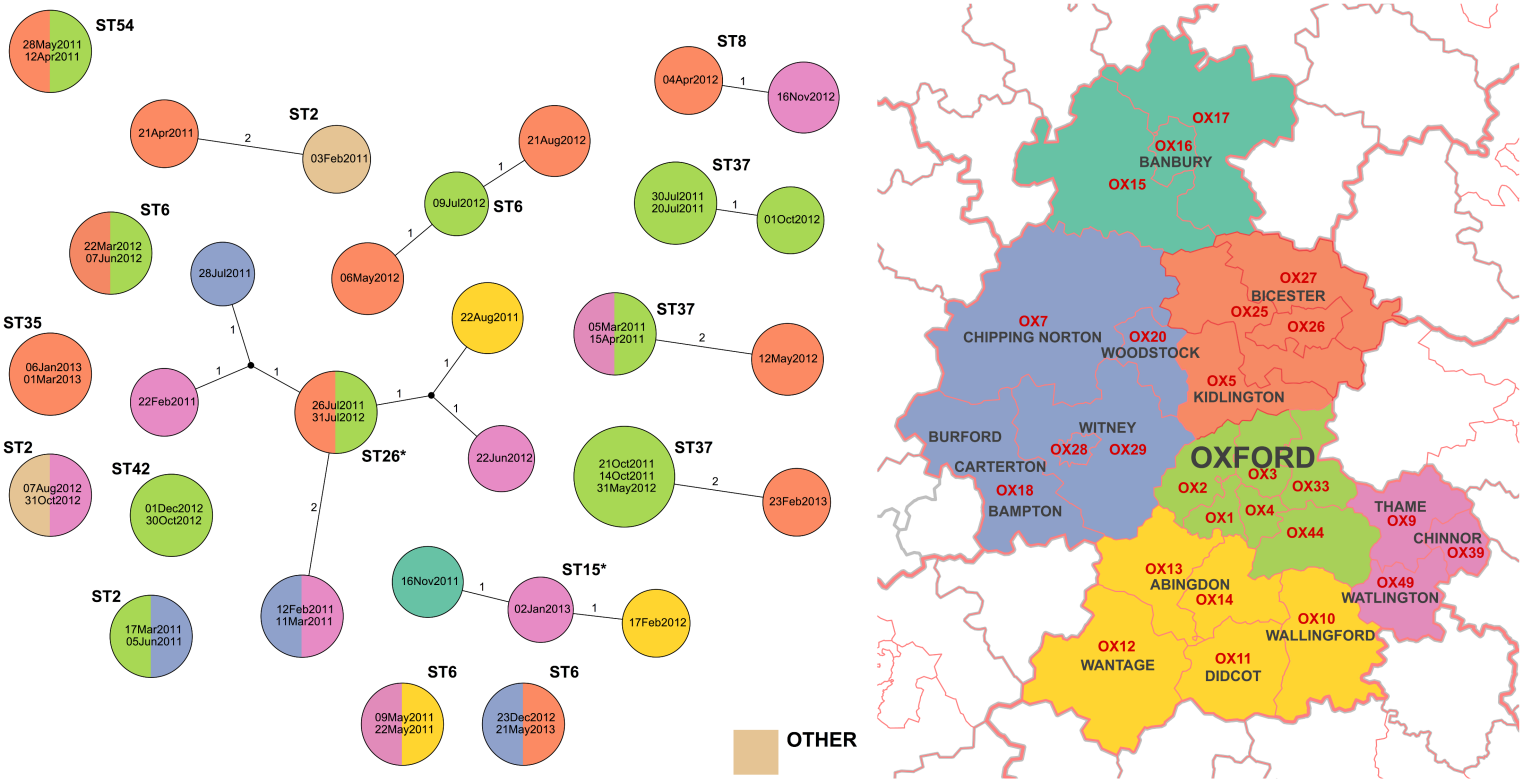
Clusters of infant isolates related within two SNVs. Each isolate is shown as a circle colored according to the infant’s home location on the map. SNVs between isolates are labeled on the connecting lines. Where indistinguishable sequences were obtained from more than one infant, the size of the circle is increased and the circle labeled with each isolate’s collection date. *ST 26 and ST15 are non-toxigenic, all other STs shown are toxigenic.

**Fig 4:**
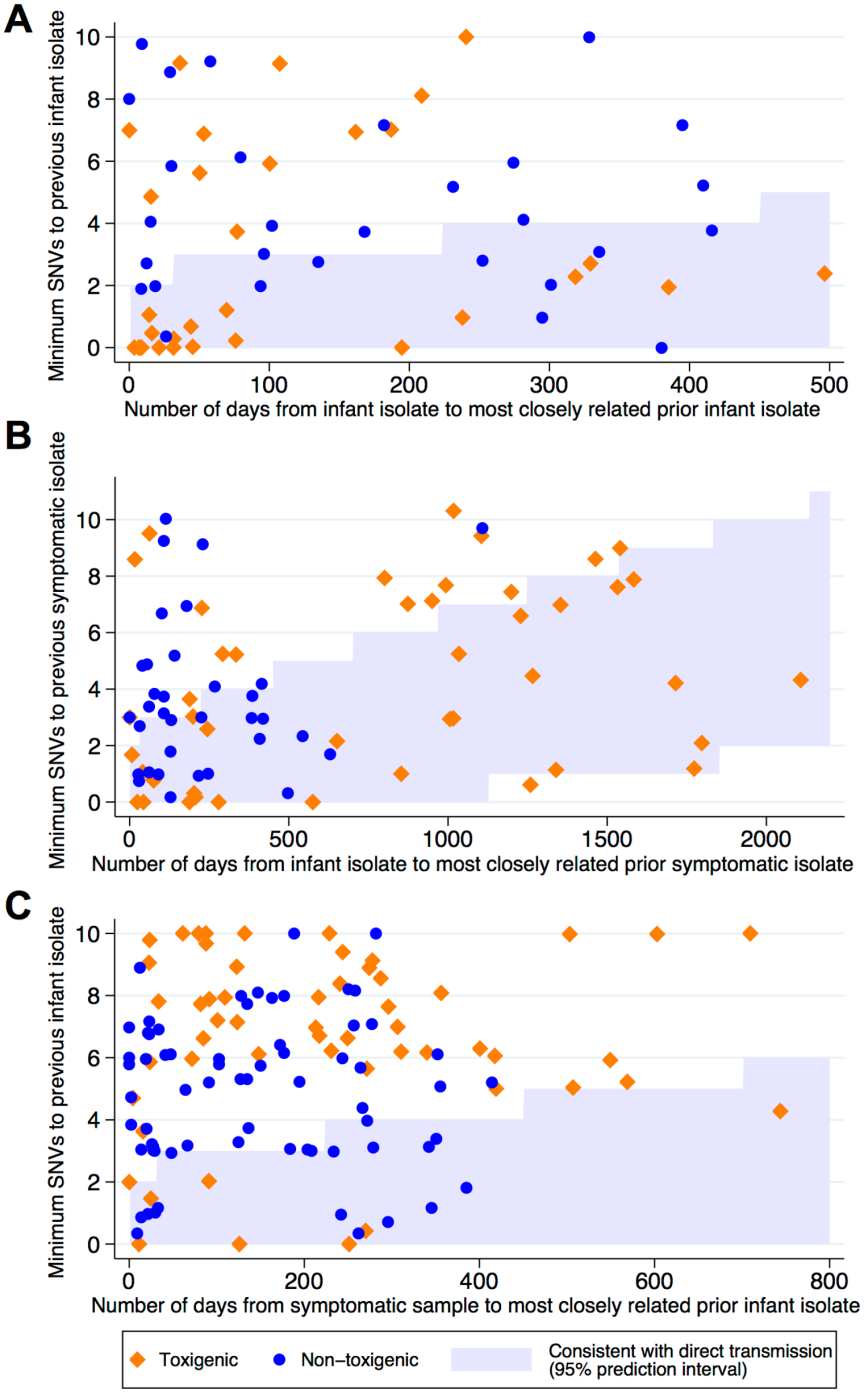
Comparison of the number of SNVs and elapsed time between *C*. *difficile* isolates (toxigenic and non-toxigenic) related within ten SNVs in Oxfordshire. Panel A compares samples from infants to all prior isolates from infants i.e. whether infants might plausibly be transmitting to other infants; the most closely related prior sequence is plotted. Panel B compares infants with prior symptomatic patients, i.e. shows whether symptomatic patients might plausibly be a source of infant carriage or colonization. Panel C compares infants with subsequent symptomatic patient sequences, i.e. shows where infant carriage is a potential source of subsequent symptomatic infection. Compatibility with transmission from source to recipient (either direct or via one or more intermediates) is determined by evolutionary rates (x-axis scales differ); the 95% prediction interval for compatibility with transmission is represented by the shaded blue areas.

The first sequence from each infant colonization or carriage episode was compared with all previous infant sequences. 13/158(8%) were genetically indistinguishable (zero SNVs) from ≥1 prior sequences in another infant and 25/158(16%) were within two SNVs, i.e. sufficiently closely related to be compatible with recent direct transmission/acquisition from a common source, given rates of *C*. *difficile* evolution and within-host diversity[14]. There was no evidence of geographic clustering of related isolates within Oxfordshire(Fig 3). 60/158(38%) infant isolates were within 0–10 SNVs of another infant strain(s)(Fig 4A), i.e. related by a common ancestor during or shortly before the study (~5 years evolution).

### Genetic relatedness of Oxfordshire isolates

Sequences from infant colonization or carriage were compared with prior sequences from symptomatic Oxfordshire patients from September 2006 onwards, to evaluate whether symptomatic patients were a plausible source of infant *C*. *difficile* colonization or carriage. Nine (6%) of 158 infant colonization or carriage episodes were genetically indistinguishable from a previous sequence from a symptomatic patient, and 29(18%) and 79(50%) were within two and ten SNVs, respectively(Fig 4B).

Excluding three infant colonization or carriage episodes pre-January 2011, infant colonization or carriage isolates from January 2011 were compared with subsequent strains from symptomatic patients through September 2013, i.e. exploring if CDI cases could have arisen from colonized/carrier infants or a shared source. Of 839 symptomatic patients, six(1%) had a genetically indistinguishable prior infant isolate, and 17(2%) and 122(15%) had a prior infant strain within two and ten SNVs, respectively(Fig 4C, S3 and S4 Figs). Overall 13% of Oxfordshire symptomatic patients were within ten SNVs of the Oxfordshire infant strains.

## Discussion

In this large study of *C*. *difficile* isolates from 1,332 fecal samples and 338 infants we demonstrate close genetic overlap between strains present in colonized, predominantly healthy infants and those causing CDI. Similar to previous studies, around one-third of infants carried *C*. *difficile*. However, despite the wide diversity of *C*. *difficile* in symptomatic patients[14], half of the *C*. *difficile* carried by infants was sufficiently genetically related to at least one prior symptomatic patient (predominantly adult CDIs) from Oxfordshire to be compatible with a common source in the previous five years (within ten SNVs). Furthermore, despite only ~1% of all Oxfordshire infants being enrolled into the study[24], 2% of symptomatic patients (again, predominantly adult CDIs) had a sufficiently genetically similar prior infant colonization or carriage isolate to support recent direct/indirect transmission, and 15% of symptomatic patients shared a common source with an infant strain with five years. This overlap could represent a shared reservoir or direct transmission between infants and CDI cases, although the temporal-spatial overlap between predominantly healthy children and patients with CDI might be expected to be reasonably limited. This overlap appears to be predominantly restricted to a few STs (toxigenic ST2, ST8, and ST11, and non-toxigenic ST15 and ST26).

As in previous, smaller, published surveys of asymptomatic children[5, 6], ST1(NAP1/BI/027) was notably absent in this population group, suggesting healthcare exposure may be particularly important for its transmission in this region. This is supported by the relative rarity of this strain in studies of asymptomatic populations in the community and of community-associated CDI cases outside of North America[5, 6, 25–31]. Molecular studies of isolates colonizing infants in the USA would be warranted, in order to ascertain the epidemiology of infant colonization and carriage in a setting with high rates of both community-associated and healthcare-associated CDI attributable to ST1(027)[29].

Independent factors associated with infant *C*. *difficile* colonization or acquisition identified here suggest that many infant-associated lineages may be transmitted from the environment. Although immunity and the microbiome change with age, increasing age also involves increasing exposure to the wider environment and other children. Exclusive breastfeeding likely reduces the risk of ingestion of *C*. *difficile* as well as potentially exerting immunological effects on the infant microbiome[32], and exposure to pets and childcare workers/institutions, mode of birth, antibiotic consumption and pre-existing colonization all reflect the interplay between predisposition to colonization and varying environmental exposure, given that *C*. *difficile* is present in many environmental substrates, including water, soil and food[33–35]. Pets are relatively commonly colonized with *C*. *difficile*, although it is unclear whether this just reflects wider household *C*. *difficile* colonization[36].

This study has several potential limitations. Firstly, we used ethanol shock/selective culture without enrichment to identify *C*. *difficile*, which may have missed low-grade colonization (<10^2^ CFU/ml)[37]. An alternative, approach might have been a concomitant PCR-based assay to confirm the proportion of positive samples for which a *C*. *difficile* isolate was retrievable. Nevertheless, the rates of infant colonization observed in our study are not dissimilar to those published elsewhere[4]. We may also have missed mixed infections by following up only single isolates from each case; the prevalence of mixed infection or colonization ranges from 3–13%[38, 39]. Secondly, our study of infant *C*. *difficile* was undertaken when ribotype-027/ST1 was a declining cause of CDI in our region and the UK[14, 40]. Nevertheless, the absence of ST1(027) in this study was consistent with a previous smaller study in Oxfordshire infants(November 2008–2009)[5], when ST1(027) caused ~30% CDI cases, but was not found in 128 infants (95% CI:0–2.8%). Thirdly, we did not record the number of infants approached for participation or reasons for refusal, perhaps resulting in selection bias. However, infants were recruited from 19/30 Oxfordshire postal districts, including both urban and rural, and affluent and deprived areas. The fact that only ~1% of infants were sampled suggests that actual proportion of genetically related isolates could be higher; however, sampling was done without knowledge of lineage and the lineage distributions identified should therefore be robust. Finally, our study included fewer symptomatic infants than originally planned, and a modest number of infants overall, meaning power particularly to detect interactions was limited.

This study confirms factors associated with infant *C*. *difficile* colonization identified in previous studies, and identifies pet dogs as a novel risk factor. In addition, systemic antibiotics represent a specific risk for colonization with toxigenic *C*. *difficile*. Strain switching during longitudinal carriage is not uncommon in infants. The study also adds to increasing data that *C*. *difficile* transmission may be more heterogeneous than previously appreciated. Over 30% of patients with community-associated CDI do not have typical risk factors, in particular recent antibiotics or hospitalisation[41]. Our data showing frequent links between some strains from predominantly healthy, non-hospitalized infants and CDI isolates supports common community reservoirs of *C*. *difficile*; this remains to be investigated in a large and targeted study.

## Supporting information

S1 Fig Effect of age on *Clostridium difficile* colonization prevalence at enrolment. Panel A shows adjusted odds ratios (versus 6 months) for carriage with any strains, panel B non-toxigenic strains, panel C toxigenic strains from the multivariable fractional polynomial models.
(PDF)

S2 Fig *Clostridium difficile* evolution and within host diversity in infants and symptomatic patients. Comparisons of first and last sequences from 69 infants under two years are shown as blue circles, and comparisons of first and last sequences from 145 symptomatic patients are shown as red diamonds. The expected number of SNVs is shown as a dotted line, 95% confidence intervals shaded. There was no evidence for a difference in *C*. *difficile* evolutionary rates between symptomatic patients and infants (heterogeneity p = 0.86).
(PDF)

S3 Fig Genetic clusters containing infant sequences over time. Each genetic cluster is defined as being greater than ten SNVs different from any other Oxfordshire isolate and is assigned an arbitrary number. The STs corresponding to the major clusters are annotated.
(PDF)

S4 Fig Clusters of infant sequences related to symptomatic patients within two SNVs. Each sequence is shown as a circle labeled with the date of collection. Where indistinguishable sequences were obtained from more than one infant/symptomatic patient the size of the circle is increased and the circle labeled with each isolate’s collection date. Sequences from infant participants are suffixed with an *. Sequences from symptomatic patients are colored red, from infants green and where indistinguishable sequences were obtained from both symptomatic patients and infants yellow.
(PDF)

S1 Table Risk factors at enrolment and association with colonization by *Clostridium difficile*: Univariable logistic (all strains) and multinomial (non-toxigenic, toxigenic strains) regression. (DOCX)

S2 Table Risk factors for colonization at enrolment: Interaction between pet dogs and nutrition in multivariable logistic regression model. (DOCX)

S3 Table Association between enrolment and time-updated risk factors with acquisition of a new strain: Univariable Cox regression. (DOCX)

S4 Table Independent risk factors for time-to-acquisition of a distinct *C*. *difficile* strain in 127 infants followed longitudinally. (DOCX)

S1 File Supporting material. Additional details of methods.
(DOCX)
