# Supplementary figures and images for: Epidemiology of Clostridium difficile in infants in Oxfordshire, UK: Risk factors for colonization and carriage, and genetic overlap with regional C. difficile infection strains
Source: PLoS One. 2017 Aug 16;12(8):e0182307. doi: 10.1371/journal.pone.0182307 (PMC5559064; doi:10.1371/journal.pone.0182307)

**A**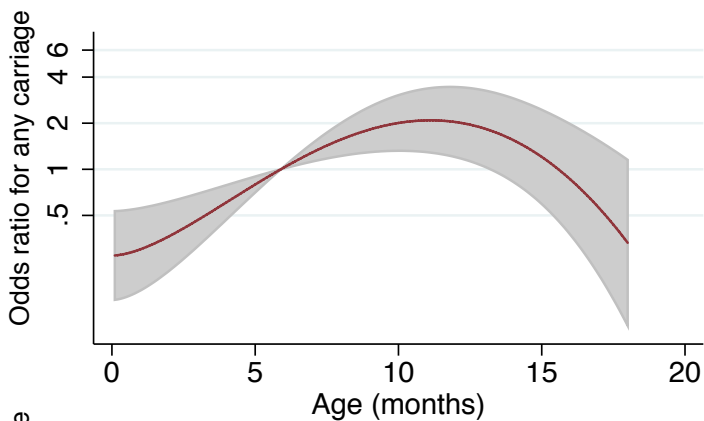**B**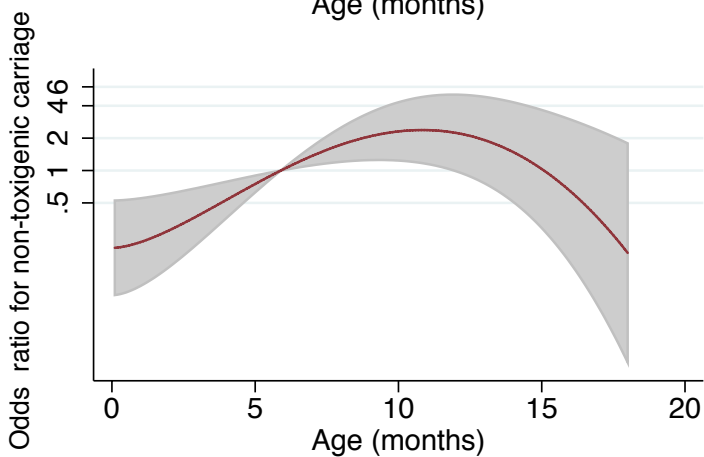**C**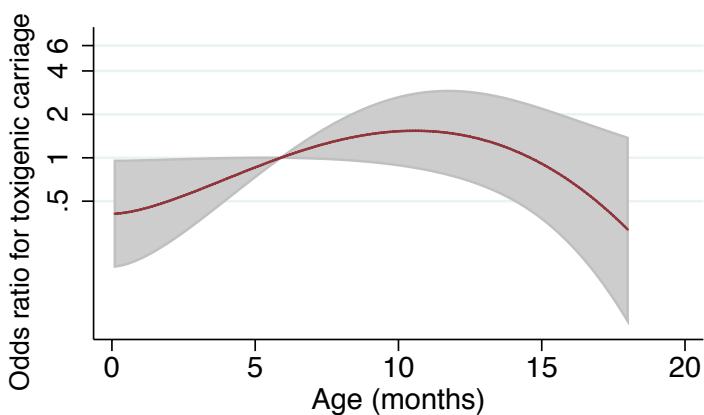

Supplement: S1 Fig — Panel A shows adjusted odds ratios (versus 6 months) for carriage with any strains, panel B non-toxigenic strains, panel C toxigenic strains from the multivariable fractional polynomial models. (PDF) [file pone.0182307.s001.pdf]

Single nucleotide variants

Time between first and last isolates, days

6  
5  
4  
3  
2  
1  
0

0

50

100

150

200

250

0

1

2

3

4

5

6

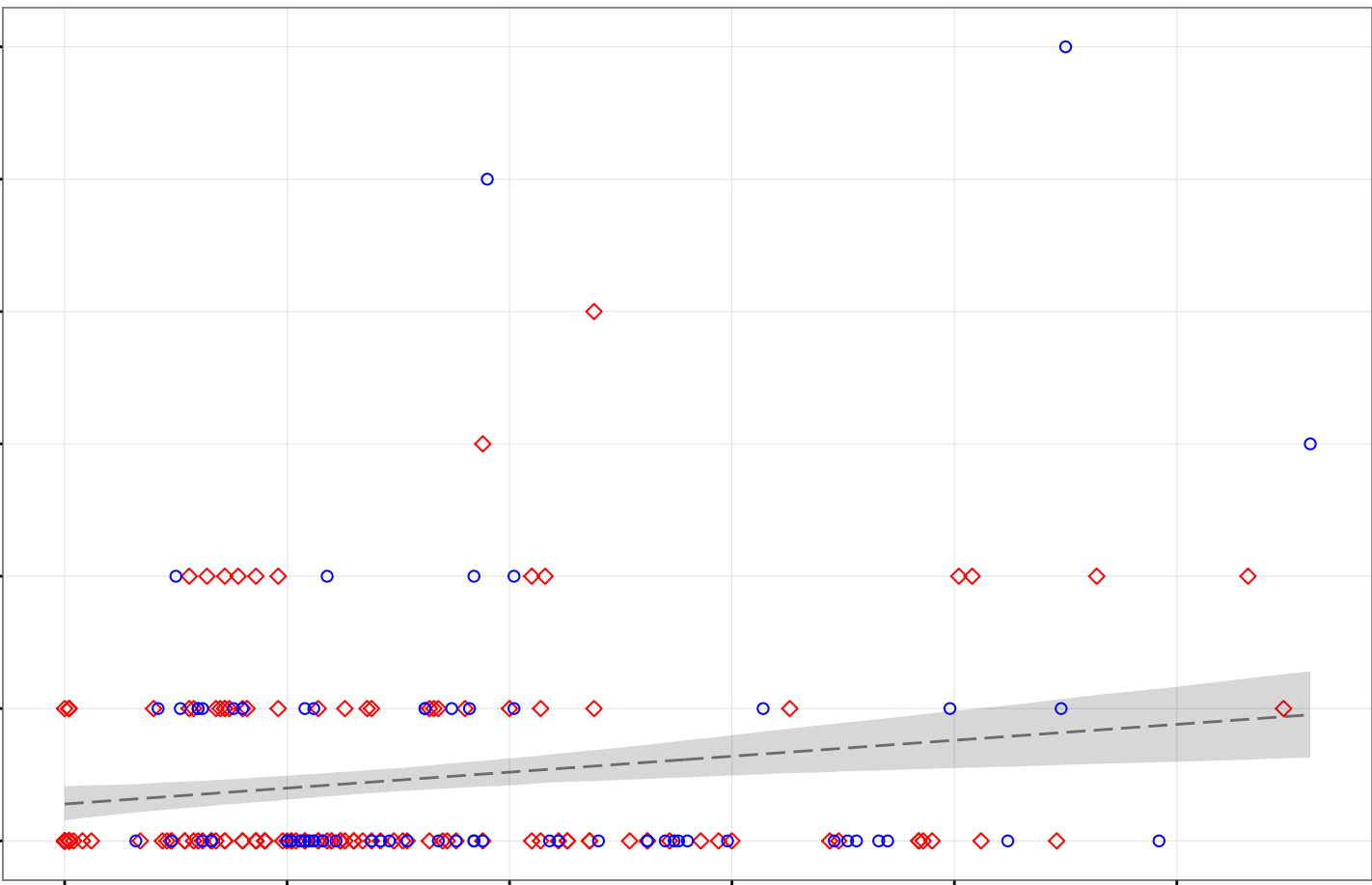

Supplement: S2 Fig — Comparisons of first and last sequences from 69 infants under two years are shown as blue circles, and comparisons of first and last sequences from 145 symptomatic patients are shown as red diamonds. The expected number of SNVs is shown as a dotted line, 95% confidence intervals shaded. There was no evidence for a difference in C. difficile evolutionary rates between symptomatic patients and infants (heterogeneity p = 0.86). (PDF) [file pone.0182307.s002.pdf]

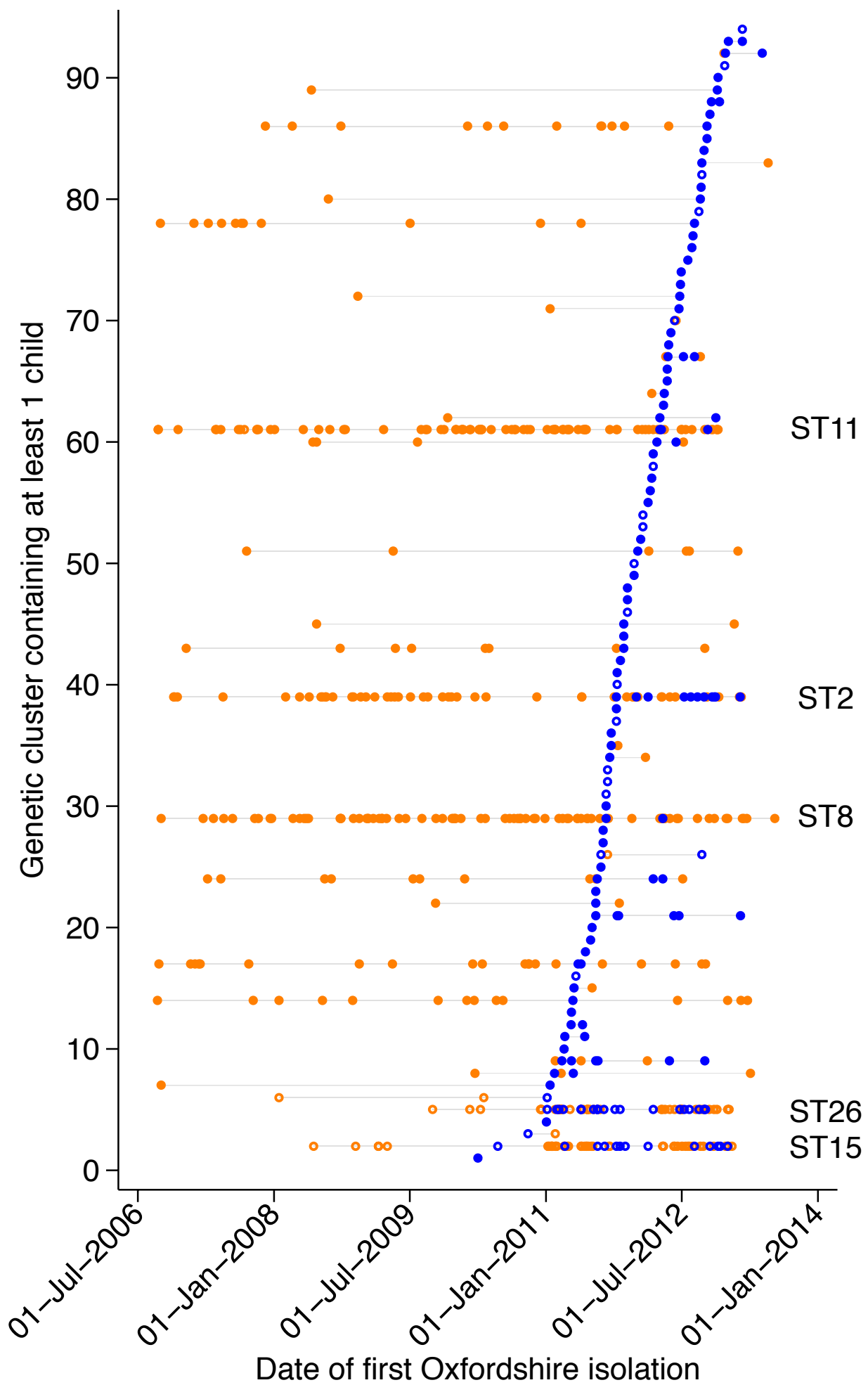

Supplement: S3 Fig — Each genetic cluster is defined as being greater than ten SNVs different from any other Oxfordshire isolate and is assigned an arbitrary number. The STs corresponding to the major clusters are annotated. (PDF) [file pone.0182307.s003.pdf]

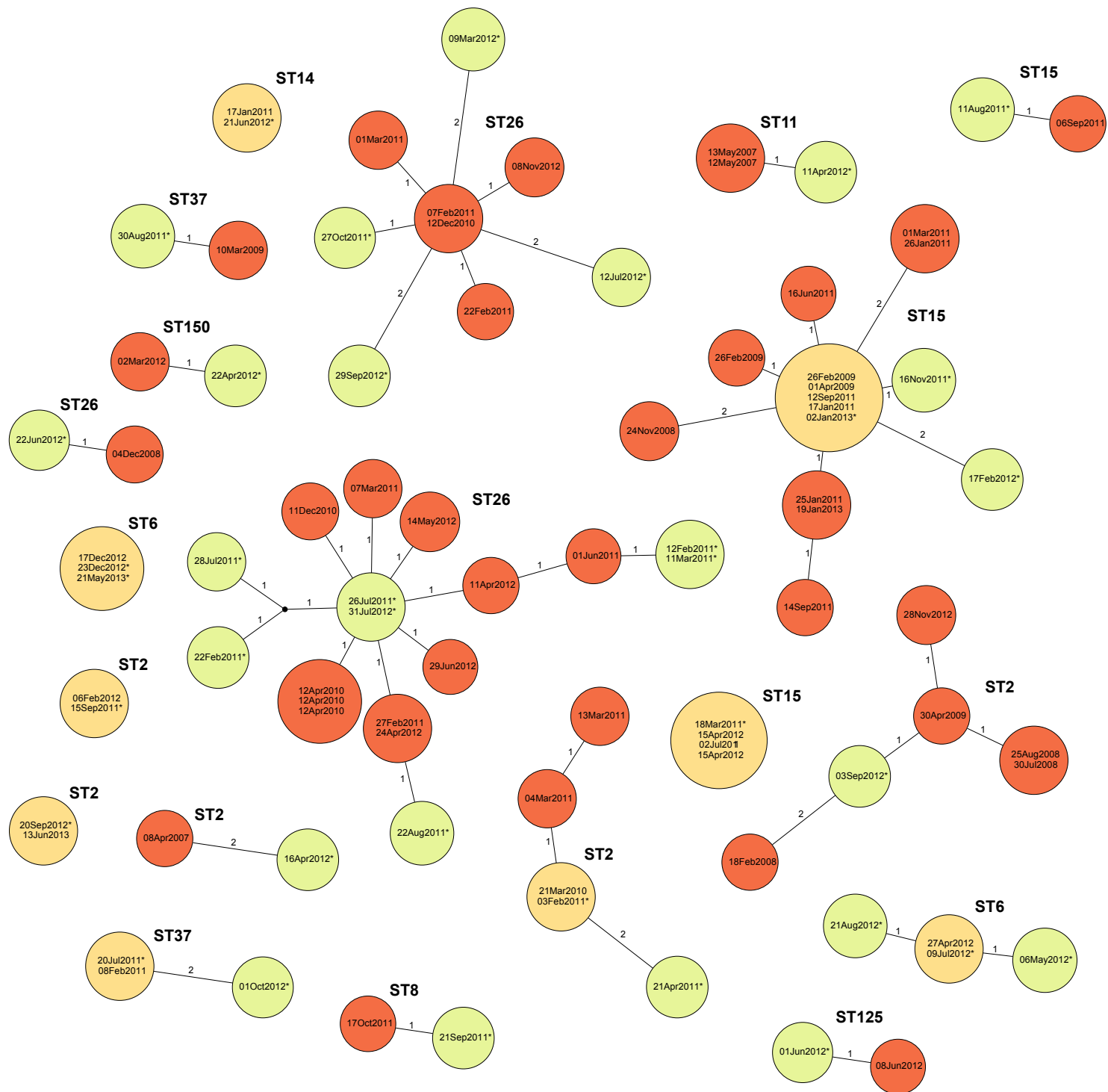

Supplement: S4 Fig — Each sequence is shown as a circle labeled with the date of collection. Where indistinguishable sequences were obtained from more than one infant/symptomatic patient the size of the circle is increased and the circle labeled with each isolate’s collection date. Sequences from infant participants are suffixed with an *. Sequences from symptomatic patients are colored red, from infants green and where indistinguishable sequences were obtained from both symptomatic patients and infants yellow. (PDF) [file pone.0182307.s004.pdf]
