# Supplementary material for: Epidemiology of Clostridium difficile in infants in Oxfordshire, UK: Risk factors for colonization and carriage, and genetic overlap with regional C. difficile infection strains
Source: PLoS One. 2017 Aug 16;12(8):e0182307. doi: 10.1371/journal.pone.0182307 (PMC5559064; doi:10.1371/journal.pone.0182307)
Supplement: S1 Table — (DOCX) [file pone.0182307.s005.docx]

|  |  | **Overall** | ***C. difficile* positive (all strains)  versus negative** | | | **Presence of non-toxigenic strains  versus *C. difficile* negative** | | | **Presence of toxigenic strains versus *C. difficile* negative** | | |
| --- | --- | --- | --- | --- | --- | --- | --- | --- | --- | --- | --- |
| **Risk factor** | **missing** | **n (% of total) or median (IQR)** | **n (% of risk factor category that are positive) or median (IQR)** | **Odds ratio (95% CI)** | **p** | **n (% of risk factor category that are non-toxigenic) or median (IQR)** | **Odds ratio (95% CI)** | **p** | **n (% of risk factor category that are toxigenic) or median (IQR)** | **Odds ratio (95% CI)** | **p** |
| Study location† | 0 |  |  |  |  |  |  |  |  |  |  |
| Breast-feeding clinic |  | 128 (38%) | 7 (5%) | 1.00 (ref) |  | 4 (3%) | 1.00 (ref) |  | 3 (2%) | 1.00 (ref) |  |
| Nurseries |  | 15 (4%) | 4 (27%) | 6.29 (1.59, 24.86) | 0.009 | 1 (7%) | 2.75 (0.28, 26.80) | 0.38 | 3 (20%) | 11.00 (1.98, 61.14) | 0.006 |
| Vaccine studies |  | 24 (7%) | 3 (13%) | 2.47 (0.59, 10.32) | 0.22 | 2 (8%) | 2.88 (0.50, 16.74) | 0.24 | 1 (4%) | 1.92 (0.19, 19.35) | 0.58 |
| Well baby clinic |  | 115 (34%) | 27 (23%) | 5.30 (2.21, 12.73) | <0.001 | 13 (11%) | 4.47 (1.41, 14.17) | 0.01 | 14 (12%) | 6.42 (1.79, 23.01) | 0.004 |
| Hospital - <48h |  | 49 (14%) | 15 (31%) | 7.63 (2.88, 20.21) | <0.001 | 5 (10%) | 4.45 (1.13, 17.48) | 0.03 | 10 (20%) | 11.86 (3.09, 45.54) | <0.001 |
| Hospital - >48h |  | 7 (2%) | 2 (29%) | 6.91 (1.13, 42.17) | 0.04 | 0 (0%) | No cases |  | 2 (29%) | 16.13 (2.18, 119.25) | 0.006 |
| Study type | 0 |  |  |  |  |  |  |  |  |  |  |
| - Cross-sectional only |  | 208 (62%) | 46 (22%) | 1.00 (ref) |  | 19 (9%) | 1.00 (ref) |  | 27 (13%) | 1.00 (ref) |  |
| - Longitudinal |  | 130 (38%) | 12 (9%) | 0.36 (0.18, 0.71) | 0.003 | 6 (5%) | 0.43 (0.17, 1.12) | 0.08 | 6 (5%) | 0.31 (0.12, 0.76) | 0.01 |
| Gender | 0 |  |  |  |  |  |  |  |  |  |  |
| - Male |  | 171 (51%) | 23 (13%) | 1.00 (ref) |  | 11 (6%) | 1.00 (ref) |  | 12 (7%) | 1.00 (ref) |  |
| - Female |  | 167 (49%) | 35 (21%) | 1.71 (0.96, 3.04) | 0.07 | 14 (8%) | 1.43 (0.63, 3.25) | 0.4 | 21 (13%) | 1.96 (0.93, 4.14) | 0.08 |
| Hospital stay after birth (days)^**^ | 0 | 2 (1, 3) | 2 (1, 3) | 0.97 (0.90, 1.05) | 0.49 | 2 (1, 3) | 0.97 (0.85, 1.09) | 0.59 | 2 (1, 3) | 0.98 (0.90, 1.07) | 0.64 |
| Overseas travel in last year | 0 | 100 (30%) | 16 (16%) | 0.89 (0.47, 1.67) | 0.71 | 5 (5%) | 0.58 (0.21, 1.61) | 0.3 | 11 (11%) | 1.17 (0.54, 2.51) | 0.69 |
| Delivery mode | 0 |  |  |  |  |  |  |  |  |  |  |
| - Normal |  | 222 (66%) | 33 (15%) | 1.00 (ref) |  | 14 (6%) | 1.00 (ref) |  | 19 (9%) | 1.00 (ref) |  |
| - Assisted |  | 48 (14%) | 11 (23%) | 1.70 (0.79, 3.67) | 0.17 | 4 (8%) | 1.46 (0.45, 4.68) | 0.53 | 7 (15%) | 1.88 (0.74, 4.80) | 0.19 |
| - Caesarean |  | 68 (20%) | 14 (21%) | 1.48 (0.74, 2.97) | 0.26 | 7 (10%) | 1.75 (0.67, 4.55) | 0.25 | 7 (10%) | 1.29 (0.51, 3.23) | 0.59 |
| Home delivery | 3 | 9 (3%) | 2 (22%) | * |  | 2 (22%) | * |  | 0 (0%) | * |  |
| Gestation (weeks)^**^ | 0 | 40 (39, 41) | 40 (39, 41) | 0.99 (0.84, 1.16) | 0.87 | 40 (39, 41) | 1.07 (0.82, 1.40) | 0.62 | 40 (39, 41) | 0.94 (0.78, 1.13) | 0.52 |
| Weight at birth (kg)^**^ | 2 | 3.4 (3.1, 3.8) | 3.4 (3.1, 3.7) | 0.80 (0.48, 1.34) | 0.41 | 3.5 (3.3, 3.8) | 1.33 (0.67, 2.64) | 0.42 | 3.2 (2.8, 3.4) | 0.54 (0.28, 1.04) | 0.07 |
| Nutrition | 0 |  |  |  |  |  |  |  |  |  |  |
| - No breastfeeding |  | 124 (37%) | 41 (33%) | 1.00 (ref) |  | 15 (12%) | 1.00 (ref) |  | 26 (21%) | 1.00 (ref) |  |
| - Mixed feeding |  | 103 (30%) | 14 (14%) | 0.32 (0.16, 0.63) | <0.001 | 8 (8%) | 0.50 (0.20, 1.23) | 0.13 | 6 (6%) | 0.22 (0.08, 0.55) | 0.001 |
| - Breastfeeding only |  | 111 (33%) | 3 (3%) | 0.06 (0.02, 0.19) | <0.001 | 2 (2%) | 0.10 (0.02, 0.46) | 0.003 | 1 (1%) | 0.03 (0.00, 0.22) | <0.001 |
| Siblings | 0 | 126 (37%) | 20 (16%) | 0.86 (0.48, 1.56) | 0.63 | 9 (7%) | 0.92 (0.39, 2.16) | 0.85 | 11 (9%) | 0.82 (0.38, 1.76) | 0.61 |
| Siblings (<2 years) | 0 | 55 (16%) | 7 (13%) | 0.66 (0.28, 1.55) | 0.34 | 2 (4%) | 0.42 (0.10, 1.84) | 0.25 | 5 (9%) | 0.86 (0.32, 2.35) | 0.77 |
| Non-parental carer - any | 0 | 78 (23%) | 23 (29%) | 2.69 (1.47, 4.91) | 0.001 | 10 (13%) | 2.73 (1.16, 6.40) | 0.02 | 13 (17%) | 2.66 (1.25, 5.67) | 0.01 |
| - Childminder | 0 | 14 (4%) | 5 (36%) | 2.84 (0.92, 8.81) | 0.07 | 5 (36%) | 7.53 (2.30, 24.59) | <0.001 | 0 (0%) | No cases |  |
| - Grandparent | 0 | 42 (12%) | 14 (33%) | 2.86 (1.40, 5.87) | 0.004 | 6 (14%) | 2.84 (1.05, 7.71) | 0.04 | 8 (19%) | 2.88 (1.19, 6.99) | 0.02 |
| - Nursery | 0 | 35 (10%) | 11 (31%) | 2.50 (1.15, 5.44) | 0.02 | 4 (11%) | 2.03 (0.64, 6.41) | 0.23 | 7 (20%) | 2.87 (1.13, 7.30) | 0.03 |
| Pets in household - Any | 0 | 149 (44%) | 37 (25%) | 2.64 (1.47, 4.75) | 0.001 | 16 (11%) | 2.67 (1.14, 6.24) | 0.02 | 21 (14%) | 2.63 (1.24, 5.55) | 0.01 |
| - Cat | 0 | 90 (27%) | 20 (22%) | 1.58 (0.86, 2.89) | 0.14 | 9 (10%) | 1.69 (0.71, 3.99) | 0.23 | 11 (12%) | 1.50 (0.69, 3.25) | 0.30 |
| - Dog | 0 | 58 (17%) | 21 (36%) | 3.73 (1.97, 7.05) | <0.001 | 9 (16%) | 3.69 (1.52, 8.97) | 0.004 | 12 (21%) | 3.75 (1.70, 8.26) | 0.001 |
| Ever taken systemic antibiotics | 0 | 83 (25%) | 23 (28%) | 0.42 (0.23, 0.76) | 0.004 | 5 (6%) | 1.09 (0.39, 3.03) | 0.87 | 18 (22%) | 0.23 (0.11, 0.48) | <0.001 |
| Time since last taken antibiotics | 1 |  |  |  |  |  |  |  |  |  |  |
| - 0-1 months ago |  | 25 (7%) | 5 (20%) | 1.41 (0.50, 3.98) | 0.51 | 0 (0%) | No cases |  | 5 (20%) | 3.05 (1.03, 9.04) | 0.04 |
| - 1-3 months ago |  | 19 (6%) | 4 (21%) | 1.51 (0.48, 4.77) | 0.48 | 1 (5%) | 0.70 (0.09, 5.58) | 0.74 | 3 (16%) | 2.44 (0.65, 9.19) | 0.19 |
| - 3-7 months ago |  | 20 (6%) | 7 (35%) | 3.05 (1.15, 8.09) | 0.03 | 1 (5%) | 0.81 (0.10, 6.50) | 0.84 | 6 (30%) | 5.64 (1.92, 16.50) | 0.002 |
| - 7+ months ago (or never) |  | 273 (81%) | 41 (15%) | 1.00 (ref) |  | 22 (8%) | 1.00 (ref) |  | 19 (7%) | 1.00 (ref) |  |
| Ever taken high c-diff-risk antibiotics | 0 |  |  |  |  |  |  |  |  |  |  |
| - Not taken |  | 291 (86%) | 46 (16%) | 1.00 (ref) |  | 22 (8%) | 1.00 (ref) |  | 24 (8%) | 1.00 (ref) |  |
| - May have taken |  | 32 (9%) | 9 (28%) | 2.08 (0.91, 4.79) | 0.08 | 2 (6%) | 0.97 (0.21, 4.38) | 0.97 | 7 (22%) | 3.11 (1.21, 7.99) | 0.02 |
| - Taken |  | 15 (4%) | 3 (20%) | 1.33 (0.36, 4.90) | 0.67 | 1 (7%) | 0.93 (0.12, 7.47) | 0.94 | 2 (13%) | 1.70 (0.36, 8.05) | 0.5 |
| Other medical problems - ever | 0 | 77 (23%) | 17 (22%) | 1.52 (0.81, 2.86) | 0.19 | 6 (8%) | 1.16 (0.44, 3.03) | 0.76 | 11 (14%) | 1.83 (0.84, 3.99) | 0.13 |
| - currently | 0 | 69 (20%) | 15 (22%) | 1.46 (0.76, 2.82) | 0.26 | 5 (7%) | 1.05 (0.38, 2.91) | 0.93 | 10 (14%) | 1.82 (0.82, 4.05) | 0.14 |
| Ever had diarrhea | 0 | 30 (9%) | 7 (23%) | 1.53 (0.62, 3.76) | 0.35 | 3 (10%) | 1.52 (0.42, 5.48) | 0.52 | 4 (13%) | 1.54 (0.50, 4.77) | 0.45 |
| Currently has diarrhea | 0 | 29 (9%) | 7 (24%) | 1.61 (0.65, 3.97) | 0.3 | 3 (10%) | 1.60 (0.44, 5.77) | 0.47 | 4 (14%) | 1.62 (0.52, 5.02) | 0.41 |
| Gastrointestinal problems - ever | 0 | 16 (5%) | 3 (19%) | 1.12 (0.31, 4.06) | 0.86 | 1 (6%) | 0.86 (0.11, 6.83) | 0.88 | 2 (13%) | 1.33 (0.29, 6.15) | 0.72 |
| - currently | 0 | 12 (4%) | 3 (25%) | 1.64 (0.43, 6.26) | 0.47 | 1 (8%) | 1.25 (0.15, 10.33) | 0.83 | 2 (17%) | 1.94 (0.40, 9.40) | 0.41 |
| Atopy - ever | 0 | 19 (6%) | 2 (11%) | 0.55 (0.12, 2.46) | 0.44 | 1 (5%) | 0.64 (0.08, 5.06) | 0.68 | 1 (5%) | 0.48 (0.06, 3.76) | 0.49 |
| - currently | 0 | 17 (5%) | 2 (12%) | 0.63 (0.14, 2.84) | 0.55 | 1 (6%) | 0.74 (0.09, 5.82) | 0.77 | 1 (6%) | 0.55 (0.07, 4.32) | 0.57 |
| No. of medical problems ever | 0 | 0 (0, 0) | 0 (0, 1) | 1.13 (0.69, 1.83) | 0.63 | 0 (0, 0) | 0.90 (0.42, 1.95) | 0.79 | 0 (0, 1) | 1.29 (0.73, 2.27) | 0.38 |
| - No. of current medical problems | 0 | 0 (0, 0) | 0 (0, 1) | 1.15 (0.69, 1.94) | 0.59 | 0 (0, 0) | 0.86 (0.37, 2.04) | 0.74 | 0 (0, 1) | 1.36 (0.74, 2.50) | 0.32 |
| Currently on medication | 0 | 36 (11%) | 6 (17%) | 0.96 (0.38, 2.43) | 0.93 | 2 (6%) | 0.72 (0.16, 3.23) | 0.67 | 4 (11%) | 1.15 (0.38, 3.49) | 0.81 |
| Currently on antibiotics | 0 | 3 (1%) | 1 (33%) | * |  | 0 (0%) | * |  | 1 (33%) | * |  |
| Currently on gastrointestinal medication | 0 | 19 (6%) | 3 (16%) | 0.90 (0.25, 3.19) | 0.87 | 1 (5%) | 0.69 (0.09, 5.41) | 0.72 | 2 (11%) | 1.06 (0.23, 4.85) | 0.94 |
| Currently on other medications | 0 | 16 (5%) | 2 (13%) | 0.68 (0.15, 3.07) | 0.61 | 1 (6%) | 0.79 (0.10, 6.28) | 0.83 | 1 (6%) | 0.59 (0.08, 4.67) | 0.62 |
| Healthcare worker in family | 0 | 59 (17%) | 9 (15%) | 0.84 (0.39, 1.83) | 0.67 | 5 (8%) | 1.15 (0.41, 3.21) | 0.79 | 4 (7%) | 0.63 (0.21, 1.89) | 0.41 |
| Admitted to hospital - ever | 0 | 61 (18%) | 19 (31%) | 0.36 (0.19, 0.69) | 0.002 | 6 (10%) | 0.56 (0.21, 1.48) | 0.24 | 13 (21%) | 0.27 (0.13, 0.59) | <0.001 |
| - currently† | 0 | 56 (17%) | 17 (30%) | 2.56 (1.33, 4.95) | 0.005 | 5 (9%) | 1.54 (0.55, 4.36) | 0.41 | 12 (21%) | 3.53 (1.61, 7.75) | 0.002 |
| No. of times admitted to hospital | 0 | 0 (0, 0) | 0 (0, 1) | 1.68 (1.17, 2.41) | 0.005 | 0 (0, 0) | 1.31 (0.75, 2.30) | 0.35 | 0 (0, 1) | 1.86 (1.25, 2.76) | 0.002 |
| Total days in hospital | 0 | 0 (0, 0) | 0 (0, 2) | 1.01 (0.99, 1.04) | 0.38 | 0 (0, 0) | 0.99 (0.91, 1.07) | 0.74 | 0 (0, 2) | 1.02 (0.99, 1.04) | 0.19 |
| Time since last admitted to hospital | 2 |  |  |  |  |  |  |  |  |  |  |
| - 0-1 months ago |  | 34 (10%) | 12 (35%) | 3.25 (1.49, 7.06) | 0.003 | 4 (12%) | 2.22 (0.70, 7.07) | 0.18 | 8 (24%) | 4.23 (1.68, 10.64) | 0.002 |
| - 1-3 months ago |  | 8 (2%) | 1 (13%) | 0.85 (0.10, 7.09) | 0.88 | 0 (0%) | No cases |  | 1 (13%) | 1.66 (0.19, 14.14) | 0.64 |
| - 3-7 months ago |  | 9 (3%) | 4 (44%) | 4.76 (1.23, 18.47) | 0.02 | 1 (11%) | 2.44 (0.27, 21.91) | 0.43 | 3 (33%) | 6.97 (1.56, 31.22) | 0.01 |
| - 7+ months ago (or never) |  | 285 (84%) | 41 (14%) | 1.00 (ref) |  | 20 (7%) | 1.00 (ref) |  | 21 (7%) | 1.00 (ref) |  |
| Stools currently more frequent/looser than normal | 0 | 61 (18%) | 17 (28%) | 2.22 (1.16, 4.26) | 0.02 | 5 (8%) | 1.34 (0.48, 3.76) | 0.58 | 12 (20%) | 3.06 (1.41, 6.68) | 0.005 |
| Age (months) | 0 | 4.4 (1.6, 8.7) | 8.0 (5.2, 11.8) | 1.09 (1.04, 1.14) | <0.001 | 8.7 (6.1, 10.7) | 1.07 (1.01, 1.14) | 0.02 | 7.6 (5.2, 12.6) | 1.09 (1.03, 1.15) | 0.003 |
| Age (months) - truncated at 95th percentile | 0 | 4.4 (1.6, 8.7) | 8.0 (5.2, 11.8) | 1.10 (1.05, 1.16) | <0.001 | 8.7 (6.1, 10.7) | 1.09 (1.02, 1.17) | 0.01 | 7.6 (5.2, 12.6) | 1.10 (1.04, 1.17) | 0.001 |

^*^ only factors with >3% prevalence considered in models

^**^ Odds of *C. difficile* colonization also modelled using fractional polynomials (Hospital stay after birth: p=0.28, Gestation: p=0.75, Weight at birth: p=0.24)

† Omitted from multivariate model selection due to collinearity

Note: percentages are of non-missing values
