# Supplementary material for: Epidemiology of Clostridium difficile in infants in Oxfordshire, UK: Risk factors for colonization and carriage, and genetic overlap with regional C. difficile infection strains
Source: PLoS One. 2017 Aug 16;12(8):e0182307. doi: 10.1371/journal.pone.0182307 (PMC5559064; doi:10.1371/journal.pone.0182307)
Supplement: S2 Table — (DOCX) [file pone.0182307.s006.docx]

|  |  | ***C. difficile* positive (all strains)**  **n=58** | | |
| --- | --- | --- | --- | --- |
| **Risk factor** | **N** | **n (row %)** | **Odds ratio (95% CI)** | **p** |
| **No pet dog** |  |  |  |  |
| **Nutrition** - no breast | 94 | 28 (30%) | 1.00 (ref) |  |
| - Mixed | 89 | 8 (9%) | 0.23 (0.09, 0.55) | 0.001 |
| - Breast only | 97 | 1 (1%) | 0.05 (0.01, 0.43) | 0.006 |
| **Pet dog** |  |  |  |  |
| **Nutrition** - no breast | 30 | 13 (43%) | 1.41 (0.57, 3.49) | 0.45 |
| - Mixed | 14 | 6 (43%) | 2.65 (0.75, 9.39) | 0.13 |
| - Breast only | 14 | 2 (14%) | 0.71 (0.13, 3.72) | 0.68 |

Note: Interaction p=0.02. Also adjusted for age which had very similar effects to those shown in Table 1 and S2 Fig.
