# Supplementary material for: Epidemiology of Clostridium difficile in infants in Oxfordshire, UK: Risk factors for colonization and carriage, and genetic overlap with regional C. difficile infection strains
Source: PLoS One. 2017 Aug 16;12(8):e0182307. doi: 10.1371/journal.pone.0182307 (PMC5559064; doi:10.1371/journal.pone.0182307)
Supplement: S3 Table — (DOCX) [file pone.0182307.s007.docx]

|  |  | **Overall** | ***C. difficile* acquisition (all strains)** | | | **Acquisition of non-toxigenic strains** | | | **Acquisition of toxigenic strains** | | |
| --- | --- | --- | --- | --- | --- | --- | --- | --- | --- | --- | --- |
| **Risk factor** | **missing** | **n (col %)** | **n (row %) at acquisition** | **Hazard ratio (95% CI)** | **p** | **n (row %) at acquisition** | **Hazard ratio (95% CI)** | **p** | **n (row %) at acquisition** | **Hazard ratio (95% CI)** | **p** |
| More frequent or looser stools at this visit | 0 | 67 (7%) | 4 (6%) | 1.52 (0.55, 4.20) | 0.42 | 0 (0%) | No cases |  | 5 (7%) | 2.30 (0.91, 5.82) | 0.08 |
| Pets in household - Any | 0 | 445 (45%) | 34 (8%) | 1.24 (0.79, 1.96) | 0.35 | 11 (2%) | 0.88 (0.41, 1.89) | 0.74 | 28 (6%) | 1.25 (0.75, 2.08) | 0.39 |
| - Cat | 0 | 261 (26%) | 21 (8%) | 1.36 (0.82, 2.25) | 0.24 | 5 (2%) | 0.67 (0.26, 1.78) | 0.43 | 18 (7%) | 1.47 (0.85, 2.56) | 0.17 |
| - Chicken | 0 | 29 (3%) | 2 (7%) | 2.06 (0.50, 8.43) | 0.31 | 1 (3%) | 1.79 (0.24, 13.30) | 0.57 | 1 (3%) | 0.74 (0.10, 5.36) | 0.77 |
| - Dog | 0 | 157 (16%) | 12 (8%) | 0.97 (0.52, 1.80) | 0.93 | 3 (2%) | 0.55 (0.17, 1.84) | 0.33 | 11 (7%) | 1.20 (0.62, 2.31) | 0.58 |
| Non-parental carer - Any | 0 | 243 (25%) | 22 (9%) | 1.31 (0.78, 2.19) | 0.3 | 12 (5%) | 1.70 (0.77, 3.75) | 0.19 | 17 (7%) | 1.09 (0.61, 1.96) | 0.77 |
| - Childminder | 0 | 53 (5%) | 5 (9%) | 1.58 (0.63, 3.92) | 0.33 | 5 (9%) | 3.42 (1.28, 9.16) | 0.01 | 4 (8%) | 0.95 (0.34, 2.67) | 0.93 |
| - Grandparent | 0 | 122 (12%) | 11 (9%) | 1.08 (0.56, 2.08) | 0.82 | 6 (5%) | 1.42 (0.56, 3.60) | 0.46 | 6 (5%) | 0.68 (0.29, 1.59) | 0.37 |
| - Nursery | 0 | 216 (22%) | 24 (11%) | 1.69 (1.02, 2.80) | 0.04 | 7 (3%) | 0.87 (0.36, 2.11) | 0.76 | 23 (11%) | 2.20 (1.29, 3.75) | 0.004 |
| Nutrition | 7 |  |  |  |  |  |  |  |  |  |  |
| - no breastfeeding |  | 395 (40%) | 38 (10%) |  |  | 13 (3%) |  |  | 34 (9%) |  |  |
| - mixed feeding |  | 452 (46%) | 36 (8%) | 0.51 (0.32, 0.82) | 0.005 | 14 (3%) | 0.97 (0.45, 2.06) | 0.93 | 26 (6%) | 0.47 (0.28, 0.78) | 0.004 |
| - breastfeeding only |  | 131 (13%) | 1 (1%) | 0.07 (0.01, 0.50) | 0.008 | 1 (1%) | 0.39 (0.05, 3.32) | 0.39 | 0 (0%) | No cases | 1 |
| Meals include meat - Any | 2 | 338 (34%) | 33 (10%) | 1.25 (0.77, 2.04) | 0.36 | 13 (4%) | 1.23 (0.57, 2.69) | 0.6 | 26 (8%) | 1.15 (0.67, 1.97) | 0.6 |
| - Poultry | 254 | 60 (6%) | 6 (10%) | 1.13 (0.47, 2.74) | 0.78 | 1 (2%) | 0.53 (0.07, 4.08) | 0.54 | 6 (10%) | 1.46 (0.60, 3.56) | 0.41 |
| - Beef | 261 | 28 (3%) | 6 (21%) | * |  | 2 (7%) | * |  | 5 (18%) | * |  |
| - Pork | 263 | 14 (1%) | 0 (0%) | * |  | 0 (0%) | * |  | 0 (0%) | * |  |
| - Fish | 176 | 121 (12%) | 9 (7%) | 1.19 (0.58, 2.48) | 0.63 | 3 (2%) | 0.97 (0.28, 3.36) | 0.96 | 9 (7%) | 1.16 (0.55, 2.47) | 0.69 |
| - Unknown | 2 | 264 (27%) | 25 (9%) | 1.14 (0.69, 1.89) | 0.6 | 10 (4%) | 1.08 (0.48, 2.41) | 0.85 | 20 (8%) | 1.08 (0.62, 1.89) | 0.79 |
| Medication since last sample - Any | 0 | 305 (31%) | 28 (9%) | 1.10 (0.68, 1.78) | 0.69 | 9 (3%) | 0.91 (0.41, 2.02) | 0.81 | 23 (8%) | 1.26 (0.75, 2.12) | 0.38 |
| - antibiotics | 0 | 42 (4%) | 5 (12%) | 1.59 (0.64, 3.96) | 0.32 | 1 (2%) | 1.03 (0.14, 7.66) | 0.97 | 5 (12%) | 2.02 (0.80, 5.08) | 0.13 |
| - gastrointestinal | 0 | 42 (4%) | 3 (7%) | 1.00 (0.31, 3.19) | 0.99 | 2 (5%) | 2.03 (0.47, 8.72) | 0.34 | 2 (5%) | 0.73 (0.18, 3.01) | 0.67 |
| - other | 0 | 244 (25%) | 22 (9%) | 1.05 (0.63, 1.75) | 0.86 | 6 (2%) | 0.66 (0.26, 1.65) | 0.38 | 18 (7%) | 1.23 (0.71, 2.13) | 0.47 |
| Medical problems since last sample - Any | 2 | 236 (24%) | 9 (4%) | 1.30 (0.79, 2.15) | 0.3 | 1 (0%) | 1.48 (0.66, 3.31) | 0.34 | 9 (4%) | 1.35 (0.78, 2.36) | 0.29 |
| - diarrhea | 2 | 61 (6%) | 2 (3%) | 2.43 (1.19, 4.94) | 0.01 | 0 (0%) | 0.63 (0.08, 4.70) | 0.65 | 2 (3%) | 3.04 (1.48, 6.27) | 0.003 |
| - gastrointestinal | 2 | 26 (3%) | 2 (8%) | * |  | 2 (8%) | * |  | 1 (4%) | * |  |
| - atopy | 2 | 31 (3%) | 22 (71%) | 0.78 (0.19, 3.20) | 0.73 | 9 (29%) | 2.09 (0.49, 8.96) | 0.32 | 18 (58%) | 0.53 (0.07, 3.84) | 0.53 |
| Medical visit since last sample | 2 | 186 (19%) | 17 (9%) | 1.33 (0.77, 2.29) | 0.31 | 6 (3%) | 1.05 (0.42, 2.63) | 0.91 | 14 (8%) | 1.36 (0.75, 2.48) | 0.31 |
| Been in hospital since last sample | 2 | 22 (2%) | 0 (0%) | * |  | 0 (0%) | * |  | 0 (0%) | * |  |
| Healthcare worker in family | 0 | 138 (14%) | 11 (8%) | 1.41 (0.74, 2.68) | 0.29 | 3 (2%) | 0.63 (0.18, 2.13) | 0.45 | 10 (7%) | 1.58 (0.80, 3.12) | 0.19 |
| Overseas travel | 2 | 84 (9%) | 9 (11%) | 0.82 (0.39, 1.73) | 0.61 | 5 (6%) | 1.47 (0.55, 3.93) | 0.45 | 6 (7%) | 0.82 (0.35, 1.91) | 0.64 |
| Colonized with *C-difficile* in the prior sample (any type) | 0 | 270 (27%) | 2 (1%) | 0.33 (0.08, 1.33) | 0.12 | 5 (2%) | 0.48 (0.18, 1.29) | 0.15 | 5 (2%) | 0.40 (0.14, 1.13) | 0.08 |

Note: n (%) refers to monthly intervals in the Cox regression. Time to first new strain, first new non-toxigenic strain, and first new toxigenic strain considered separately.
