# Supplementary material for: Epidemiology of Clostridium difficile in infants in Oxfordshire, UK: Risk factors for colonization and carriage, and genetic overlap with regional C. difficile infection strains
Source: PLoS One. 2017 Aug 16;12(8):e0182307. doi: 10.1371/journal.pone.0182307 (PMC5559064; doi:10.1371/journal.pone.0182307)
Supplement: S4 Table — (DOCX) [file pone.0182307.s008.docx]

|  | **Acquisition of any new strain (N = 77)** | | **Heterogeneity of effect for non-toxigenic versus toxigenic acquisition** | **Acquisition of new non-toxigenic strain (N = 28)** | | **Acquisition of new toxigenic strain (N = 62)** | |
| --- | --- | --- | --- | --- | --- | --- | --- |
| **Risk factor** | **Hazard ratio (95% CI)** | **p** | **p** | **Hazard ratio (95% CI)** | **p** | **Hazard ratio (95% CI)** | **p** |
| **Age at enrolment** (per month older) | 1.23 (1.06, 1.42) | 0.008 | 0.26 | 1.14 (0.88, 1.48) | 0.33 | 1.35 (1.17, 1.56) | <0.001 |
| **Delivery mode** |  |  |  |  |  |  |  |
| Normal | 1.00 (ref) |  |  | 1.00 (ref) |  | 1.00 (ref) |  |
| Assisted | 1.55 (0.84, 2.87) | 0.16 | 0.98 | 1.64 (0.55, 4.92) | 0.38 | 1.66 (0.87, 3.17) | 0.12 |
| Caesarean | 2.60 (1.42, 4.79) | 0.002 | 0.39 | 3.39 (1.41, 8.14) | 0.006 | 2.05 (1.03, 4.10) | 0.04 |
| **Nutrition** |  |  |  |  |  |  |  |
| No breastfeeding | 1.00 (ref) |  |  | 1.00 (ref) |  | 1.00 (ref) |  |
| Mixed feeding | 0.49 (0.30, 0.79) | 0.003 | 0.27 | 0.74 (0.33, 1.70) | 0.48 | 0.43 (0.26, 0.70) | 0.001 |
| Breastfeeding only | 0.10 (0.01, 0.75) | 0.03 | - | 0.44 (0.05, 3.73) | 0.45 | No cases | - |
| **Previous sample positive with different strain** | 0.19 (0.04, 0.79) | 0.02 | 0.99 | 0.26 (0.09, 0.70) | 0.008 | 0.25 (0.08, 0.85) | 0.03 |
| **Diarrhea since the last visit** | 2.42 (1.17, 4.97) | 0.02 | 0.06 | 0.58 (0.12, 2.74) | 0.50 | 3.29 (1.75, 6.19) | <0.001 |
| **Pet cat*** | Not selected in model | | 0.08 | 0.65 (0.23, 1.82) | 0.41 | 1.96 (1.09, 3.54) | 0.03 |
| **Childminder** | Not selected in model | | 0.02 | 3.92 (1.54, 9.95) | 0.004 | 0.57 (0.18, 1.85) | 0.35 |
| **Nursery** | Not selected in model | | 0.23 | 1.00 (0.42, 2.37) | 0.997 | 1.86 (1.15, 2.99) | 0.01 |

* Although pet cats were identified in the toxigenic model using backwards elimination, a similar model fit and effect was found when substituting cats with dogs (non-toxigenic OR=0.49 p=0.21, toxigenic OR=1.29 p=0.39 for dogs, difference in Akaike Information Criteria 758(dogs)-755(cats)=4)

Note: factors are time-updated unless indicated. Acquisition of non-toxigenic versus toxigenic strain modeled and compared using stacked Cox regression. See S3 Table for univariable results. A small number of intervals with missing data (S3 Table) were excluded from multivariable analysis.
