# Supplementary material for: Epidemiology of Clostridium difficile in infants in Oxfordshire, UK: Risk factors for colonization and carriage, and genetic overlap with regional C. difficile infection strains
Source: PLoS One. 2017 Aug 16;12(8):e0182307. doi: 10.1371/journal.pone.0182307 (PMC5559064; doi:10.1371/journal.pone.0182307)
Supplement: S1 File — Additional details of methods. (DOCX) [file pone.0182307.s009.docx]

**SUPPORTING MATERIAL**

**Supporting material**

1. **Questionnaire data**

*Data collected at baseline - missing data, data processing*

Aside from the name of previous antibiotics taken (missing in 29% of cases where an antibiotic was taken), only five questions had any missing data, comprising <1% of the answers per question. As the questions were not deemed to be sensitive and rather involved recall of past events, these records were omitted from their respective univariable analyses but included where possible in the multivariable analyses (if the factor was not included in the multivariable model) (i.e. analyses were restricted to complete cases for the relevant models).

*Data collected longitudinally - missing data, data processing*

Aside from the type of meat eaten (completely missing in <1% of responses but only partially specified in 27% of responses), only five questions on longitudinal data collection forms had any missing data, comprising <1% of the answers per question. Similarly to above, these records were omitted from their respective univariable analyses but included where possible in the multivariable analyses (if the factor was not included in the multivariable model).

27 participants were excluded from the study because of failure to return a sample or a questionnaire. Two of these did not return a questionnaire and so no information was available on them. To check that the 25 excluded participants who returned a questionnaire but no sample were similar in baseline characteristics to the included participants, we conducted Fisher’s exact tests on each categorical risk factor, and conducted nonparametric K-sample tests for the equality of medians on each numerical risk factor.  No risk factors were significantly different (p<0.05) between the two groups.

1. **Details of sequencing processing and analysis**

DNA extraction kits used included: FastDNA (MP Biomedicals, California, USA), QIAamp, (Qiagen, Hilden, Germany); and QuickGene (Fujifilm, Tokyo, Japan). A combination of standard Illumina and adapted protocols was used to produce multiplexed paired-end libraries of DNA extracts. Briefly, indexed pools of 96 samples were sequenced at the Wellcome Trust Centre for Human Genetics, Oxford, UK, using the Illumina HiSeq 2000 platform (Illumina Inc, San Diego, CA). Sequencing reads were mapped with Stampy(3) (without Burrows-Wheeler Aligner pre-mapping, using an expected substitution rate of 0.01) to the *C. difficile* reference genome 630 (Genbank: AM180355.1). Sequences were compared using single nucleotide variants (SNVs) identified with Samtools(4) mpileup with the extended base-alignment quality flag. A consensus of ≥75% was required to support a SNV, and calls were required to be homozygous under a diploid model. Only SNVs supported by five or more reads, including one in each direction, were accepted. Sequences homologous to ≥70% of the reference sequence were considered to represent *C. difficile* (all others had <4% mapped). Five of 364 sequences considered to represent *C. difficile* in the risk factor analysis (≥70% of reference sequenced mapped) had unexpectedly high GC content (38-41%, *C. difficile* 630 chromosome 29%) in their sequences and so were excluded from the genetic analysis in case this arose from contamination and might result in false variant calls.

To improve computational efficiency in identifying closely related sequences, sequences from infants within 0-20 SNVs of any other sequence were initially pooled into groups. All variable sites within each group were then identified, and a very small number of sequences with <70% of these variable sites called (either as wild type or variant, 4/364) were excluded from further analysis, as these represent possible cross-contamination amongst closely related sequences in the same sequencing batch. Within each group, variable sites that were not called (either as wild type or variant) in ≥70% of sequences were also excluded from further analysis, as such sites may represent either regions of the genome that are difficult to identify with certainty from short-read sequencing or regions that are variably present or absent. Of 234 groups of sequences analyzed, the median (interquartile range) number of variable sites in each group before adjustment was 20 (3-67), and 19 (3-52) after.

A median (IQR) [range] 85.1% (84.2-85.6%) [78.5-89.9%] of the reference genome was identified as wild type or variant in the 355 infant samples passing quality checks. As noted previously^7^, there was limited recombination between closely related samples.

1. **Details of statistical methods**

Univariable logistic regression was used to pre-select variables for multivariable analyses, using a significance threshold of p≤0.2 on all factors present in >3% of the population. Continuous variables (i.e. age, gestation, weight at birth, length of hospital stay after birth, number of medical problems, number of times admitted to hospital, length of hospital stay for admissions) were initially modelled using a linear predictor, and those not meeting the significance threshold were further modelled using fractional polynomials.

Multivariable logistic regression was used to identify independent risk factors for initial colonization with *C. difficile* using backwards elimination (exit p=0.05) on all factors present in >3% of the population with p≤0.2 univariably. Non-linearity in continuous factors was incorporated using multivariable fractional polynomials (stata mfp)(5), truncating the continuous factors at the 95^th^ percentile to avoid undue influence from outliers. Given low rates of missing data (see above), model selection used observations complete for all factors with p≤0.2 univariably. Of the eight continuous variables assessed (age, gestation, weight at birth, length of hospital stay after birth [days], number of times taken systemic antibiotics, total number days antibiotics administered, number of times admitted to hospital, length of hospital stay for admissions) three (gestation, weight at birth, length of hospital stay after birth [days]) did not meet the pre-selection threshold (including by mfp). The final model was re-fitted in “complete-cases” for the selected factors, and any additional impact from previously eliminated factors or interactions included if p<0.05.

Multivariable multinomial regression was used to differentiate between risk factors for toxigenic and non-toxigenic strains (versus no carriage) using the same model building strategy. Odds ratios for the effects of continuous factors in Tables are calculated from the best-fitting fractional polynomial function versus the reference category as shown, and are not based on categorisation.

In the longitudinally sampled infants, time to first colonization or acquisition of a new strain (defined as >20 SNVs distant from the strain at enrolment) was estimated using Kaplan-Meier, and risk factors for acquisition of any new strain, a toxigenic new strain or a non-toxigenic new strain identified using multivariable fractional polynomial time-updated Cox regression (model building as above), censoring follow-up at the last returned sample and questionnaire. Stacked regression was used to check for heterogeneity in the effects of the combined set of risk factors on acquisition of the first new toxigenic versus the first new non-toxigenic strain.

Increasing the backwards-elimination threshold to p=0.1 did not lead to any additional variables being selected for overall colonization of *C. difficile*, though for toxigenic strains, the selection of a pet dog was replaced by the more generic pet (of any kind) variable. For acquisition, increasing the elimination threshold did not lead to any changes in the variables selected for the final models.

**Supporting material references**

1. Slimings C, Riley TV. Antibiotics and hospital-acquired Clostridium difficile infection: update of systematic review and meta-analysis. J Antimicrob Chemother. 2014;69(4):881-91.

2. Brown KA, Khanafer N, Daneman N, Fisman DN. Meta-analysis of antibiotics and the risk of community-associated Clostridium difficile infection. Antimicrob Agents Chemother. 2013;57(5):2326-32.

3. Lunter G, Goodson M. Stampy: a statistical algorithm for sensitive and fast mapping of Illumina sequence reads. Genome Res. 2011;21(6):936-9.

4. Li H, Handsaker B, Wysoker A, Fennell T, Ruan J, Homer N, et al. The Sequence Alignment/Map format and SAMtools. Bioinformatics. 2009;25(16):2078-9.

5. Royston PS, W. Multivariable Model-building: A Pragmatic Approach to Regression Analysis Based on fractional Polynomials for Modeling Continuous Variables. Chichester, UK: Wiley; 2008.
